# Estimating the Impact of the COVID-19 Pandemic on Maternal and Perinatal Health Care Services in Italy: Results of a Self-Administered Survey

**DOI:** 10.3389/fpubh.2021.701638

**Published:** 2021-07-16

**Authors:** Loredana Cena, Matteo Rota, Stefano Calza, Barbara Massardi, Alice Trainini, Alberto Stefana

**Affiliations:** ^1^Observatory of Perinatal Clinical Psychology, Section of Neuroscience, Department of Clinical and Experimental Sciences, University of Brescia, Brescia, Italy; ^2^Units of Biostatistics and Biomathematics and Bioinformatics, Department of Molecular and Translational Medicine, University of Brescia, Brescia, Italy; ^3^Department of Clinical Sciences and Community Health, University of Milan, Milan, Italy

**Keywords:** health services, antenatal and postnatal healthcare services, newborn's health, women's health, public health, maternal services

## Abstract

The coronavirus disease 2019 (COVID-19) pandemic is strongly changing the way most people live their lives, and disrupting specialist healthcare systems. Such public health disruptions have resulted in significant collateral damage with particular implications for vulnerable populations, including the perinatal population. This Study aims to estimate the impact of the COVID-19 pandemic on Italian maternal and perinatal health care services. A questionnaire was designed to evaluate the COVID-19 impact on Italian maternal and perinatal healthcare facilities and their activities and provision of services from March to May 2020. The survey was completed by hospital-based and community-based Italian maternal and perinatal healthcare facilities. Most of these were located in Lombardy or Veneto (the most affected Italian regions). 70% of all facilities reported that the first wave of the COVID-19 pandemic negatively influenced the functioning of one or more aspects of the perinatal service; only 28.4% of facilities all over the country continued to provide outpatient routine visits and examinations as usual; 23.4% of facilities became understaffed during the index period due to various reasons such as ward transfer and sick leave. This is the first Italian study, and among very few international studies that describe the effects of the COVID-19 pandemic on antenatal and postnatal healthcare facilities and their provision of activities and services. Our findings confirm that healthcare systems even in high-income countries were not entirely prepared to handle such a global health emergency; indeed, specialized maternal and perinatal healthcare services have been disrupted by this global health emergency.

## Introduction

The coronavirus disease 2019 (COVID-19) pandemic quickly and widely spread from the Hubei Province in the People's Republic of China, where the virus originated in December 2019, throughout the world, starting in early 2020 (1, 2). It has extensively changed the way most people live their daily life, including interpersonal relationships and health habits (3).

Furthermore, the morbidity and mortality associated with severe acute respiratory syndrome coronavirus 2 (SARS-CoV-2) infection have put healthcare systems worldwide under great strain (4) and also disrupted a variety of general and specialist health facilities that deliver non-COVID-19 health care services (5–10). These disruptions to public health have resulted in significant collateral damage with particular implications for vulnerable populations (11–13), including the perinatal population (7, 14–16).

Italy was the second epicenter of the spread of COVID-19 (17) and major changes have been made to the provision of health services since the outbreak in March 2020 (see Table 1). The initial rapid spread of infections and the limited number of intensive care beds available posed a critical threat to the Italian national health system (18) and its workers (thousands of healthcare professionals have been infected by the virus and many of them have died) (19, 20). Healthcare facilities constituted the main source of virus outbreaks because of hospital overcrowding and the existence of asymptomatic cases of the virus (21). After this first wave of pandemic, which ended in May 2020, the Italian government implemented significant changes to the structure of the health system in order to stem the second wave (22). However, the effects were different in different regions. The severity and the mortality of COVID-19 infection in Lombardy (which was the Italian epicenter) and Veneto were higher during this first wave of pandemic than during the second wave (between October 2020 and January 2021). For instance, in Lombardy there were 16,362 deaths (47.7% of deaths in Italy) during the first wave and 15,515 deaths (18.9% of deaths in Italy) during the second and the third wave combined (23, 24). But this does not apply to the rest of Italy, where the trend in mortality was reversed: 34,260 and 38,535 deaths in the first and second wave, respectively (23, 24).

**Table 1:** Timeline of the COVID-19 pandemic in Italy.

December 31, 2019	The Wuhan Municipal Health Commission in Wuhan City, Hubei province, China, reports a cluster of pneumonia cases (including seven severe cases) of unknown etiology.
January 9, 2020	China CDC reports that a novel coronavirus (later named SARS-CoV-2, the virus causing COVID-19) had been detected as the causative agent for 15 of the 59 cases of pneumonia.
January 17, 2020	ECDC publishes its first risk assessment on the novel coronavirus.
January 22, 2020	The Italian Ministry of Health instructs a task force to coordinate a surveillance system for suspected cases and interventions in national territory.
January 30, 2020	Two Chinese tourists hospitalized for respiratory tract infection are the first confirmed cases of COVID-19 detected in Italy. The WHO declares this first outbreak of novel coronavirus a “public health emergency of international concern.”
January 31, 2020	The Italian Council of Ministers declares a national public health emergency condition.
February 21, 2020	The Italian National Institute of Health confirms the first case of local transmission of COVID-19 infection. Over the following days, the Italian authorities reported clusters of cases in several regions (Lombardy, Piedmont, Veneto etc.).
March 8–9, 2020	The Italian Council of Ministers issues a decree to install strict public health measures starting in the most affected regions (i.e., Lombardy and Veneto). These measures include social distancing and restricting movements of people within and outside the hometown, with permitted travel limited to shopping for food, going to work (only for essential services to remain operating; work from home is encouraged), or seeking medical care. All planned surgeries are postponed in order to give over intensive care beds to the treatment of COVID-19 patients.
March 11, 2020	The Director General of the WHO declares COVID-19 a “global pandemic.” The Italian Council of Ministers extends the strict containment measures at national level.
March 13, 2020	The WHO declares Europe is becoming the new epicenter of COVID-19 pandemic.
March 31, 2020	Official reports indicated 7,593 COVID-19–associated deaths and 44,773 infected individuals. The Italian Ministry of Health issues recommendations for pregnant women, women in labor, puerperal women, newborns and breastfeeding mothers.
April, 2020	Italian scientific associations in the field of perinatal medicine (e.g., FIGO and SIN) start to publish interim recommendations for management of pregnant-woman in labor, puerperal women, newborns and breastfeeding mothers during the COVID-19 pandemic.
May 4, 2020	The Italian Council of Ministers restores the freedom of movement, and other non-essential activities re-open later in the month.
May 31, 2020	The Istituto Superiore di Sanità (in collaboration with ACP, AGUI, AOGOI, FNOPO, SIAARTI, SIGO, SIMP, SIN, SIP, and TAS) publishes interim indications for pregnancy, childbirth, breastfeeding and the care of very young children 0-2 years in response to the COVID-19 emergency.

*Table adapted from the Timeline of ECDC's response to COVID-19 (available on-line at www.ecdc.europa.eu/en/covid-19/timeline-ecdc-response)*.

*ACP, Associazione Culturale Pediatri; AOGOI, Associazione Ostetrici Ginecologi Ospedalieri Italiani; AGUI, Associazione Ginecologi Universitari Italiani; China CDC, Chinese Center for Disease Control and Prevention; ECDC, European Center for Disease Prevention and Control; FIGO, International Federation of Gynecology and Obstetrics; FNOPO, Federazione Nazionale degli Ordini della Professione di Ostetrica; SIAARTI, Società Italiana di Anestesia Analgesia Rianimazione e Terapia Intensiva; SIGO, Società Italiana di Ginecologia e Ostetricia; SIMP, Società Italiana di Medicina Perinatale; SIN, Società Italiana di Neonatologia; SIP, Società Italiana di Pediatria; TAS, Tavolo Tecnico Allattamento del Ministero della Salute; WHO, World Health Organization*.

To date, COVID-19 studies in Italy have reported the disruption of services and substantial changes in the way clinical care is delivered for mental health (25), oncology (26), surgical arthroplasty (27), pediatrics (28) and many other specialist healthcare systems. However, at the time of writing, the status of the perinatal healthcare system has not yet been comprehensively or extensively investigated. Therefore, we sought to evaluate the impact of the COVID-19 outbreak and the containment measures on maternal and perinatal healthcare services in Italy.

## Methods

### Study Design and Participants

A cross-sectional survey was conducted using an online questionnaire accessible on the University of Brescia website. The questionnaire was distributed *via* an electronic link to the coordinators or representatives of 1,428 public and private maternal and perinatal healthcare centers located throughout Italy. A brief explanation of the study purpose and assurance of anonymity was outlined in the body of the email as well as on the first page of the questionnaire. Informed written consent was obtained from all respondents before data collection. The research was approved by the Ethics Committee of ASST Spedali Civili Hospital Brescia, Italy (Approval number: NP4221 24.06.2020). The questionnaire was made available for completion from June 30 to October 7, 2020. Data were collected using LimeSurvey.

### Survey Description

The survey questionnaire was specifically created for this study: to evaluate the COVID-19 impact on both antenatal and postnatal healthcare facilities and their activities and provision of services. It was designed and trialed by a team of perinatal experts who work in maternal and perinatal clinics or are regularly involved in research in this area and in the training of healthcare workers. All the experts are members of the Observatory of Perinatal Clinical Psychology (https://www.unibs.it/it/node/988), Section of Neuroscience of the Department of Clinical and Experimental Sciences (University of Brescia, Italy). Possible misinterpretations or difficulties with wording or comprehension were discussed and resolved within the core research group. The final version of the survey questionnaire included 60 general questions, 4 additional specific questions for antenatal services and 13 for intrapartum, postnatal services. Most of the questions were closed-ended, but some optional open-ended questions were used to allow respondents to express their subjective perceptions. An example of the survey questions is “How many healthcare professionals are employed in your facility?” or “Was your facility converted into COVID-19 units during the period of health emergency (March–May 2020)?” For the latter question there were three closed-ended responses: “Yes, completely,” “Yes, but only partially,” “No”.

### Statistical Analysis

Descriptive analyses were performed. Categorical variables were recorded in terms of frequency and compared across groups using the chi-square test or the Fisher's exact test, as appropriate. Analyses were performed using R version 4.0.2 (R Foundation for statistical computing, Vienna, Austria).

## Results

### Sample Characteristics

Seventy-seven Italian perinatal healthcare facilities completed the survey (response rate 5.4%). Of these, 46 were prenatal facilities, whereas 31 were intrapartum/postnatal or maternal facilities. Thirty-nine were located in Lombardy or Veneto (the most affected Italian regions), and the remaining were from the other ten regions. Twenty-five were hospital-based, whereas the remaining were community-based. The median of healthcare professionals working in the facilities was 10 (inter-quartile range = 37.7).

All the results, unless otherwise specified, did not yield significant differences between hospital- and community-based facilities, geographical areas, or antenatal and intrapartum/postnatal care.

### Services

Seventy percent of all facilities reported that the first wave of the COVID-19 pandemic negatively influenced the functioning of one or more aspects of the maternal and perinatal services. The impact of the measures taken to prevent the spread of the virus on specific activities and services provided by Italian perinatal healthcare facilities is reported in Table 2.

**Table 2:** Impact on activities and services provided by perinatal healthcare facilities in the Italian national territory.

	**Outpatients care**												**Inpatients care**					**Outpatients care**				
	**Pregnancy and antenatal care**												**Postnatal & newborn care**								
**Has the COVID-19 pandemic adversely affected specific** **activity and service?**	**Preconception interventions and infertility treatments**	**Procreative and contraceptive counseling**	**Voluntary termination of pregnancy**	**Physiological pregnancy monitoring**	**High risk or pathological pregnancy diagnostics and monitoring**	**Twin pregnancy**	**Prevention of preterm birth**	**Cesarean section planning**	**Prenatal diagnosis**	**Assisted reproductive technology**	**Psychological counseling and support**	**Birth preparation classes**	**Delivery room**	**Postpartum obstetrical ward**	**Neonatology**	**Neonatal intensive care**	**Neonatal pathology**	**Newborn postnatal health checks**	**Psychological counseling and support**	**Home visits**	**Parenting support**	**Breastfeeding support**
Yes	21.9% (*n* = 7)	18.2% (*n* = 6)	77.4% (*n* = 24)	76.2% (*n* = 32)	75.0% (*n* = 24)	61.9% (*n* = 13)	66.7% (*n* = 12)	72.7% (*n* = 16)	63.6% (*n* = 14)	11.2% (*n* = 1)	35.9% (*n* = 14)	16.3% (*n* = 7)	64.3% (*n* = 9)	46.7% (*n* = 7)	57.1% (*n* = 8)	70.0% (*n* = 7)	53.8% (*n* = 7)	42.9% (*n* = 9)	32.0% (*n* = 8)	21.0% (*n* = 4)	22.2% (*n* = 6)	33.3% (*n* = 9)
Partially	43.7% (*n* = 14)	51.5% (*n* = 17)	19.4% (*n* = 6)	21.4% (*n* = 9)	21.9% (*n* = 7)	33.3% (*n* = 7)	33.3% (*n* = 6)	27.3% (*n* = 6)	31.8% (*n* = 7)	44.4% (*n* = 4)	61.5% (*n* = 24)	60.4% (*n* = 26)	35.7% (*n* = 5)	53.3% (*n* = 8)	42.9% (*n* = 6)	30.0% (*n* = 3)	38.5% (*n* = 5)	57.1% (*n* = 12)	64.0% (*n* = 16)	58.0% (*n* = 11)	63.0% (*n* = 17)	59.3% (*n* = 16)
No	34.4% (*n* = 11)	30.3% (*n* = 10)	3.2% (*n* = 1)	2.4% (*n* = 1)	3.1% (*n* = 1)	4.8% (*n* = 1)	0.0% (*n* = 0)	0.0% (*n* = 0)	5.6% (*n* = 1)	44.4% (*n* = 4)	2.6% (*n* = 1)	23.3% (*n* = 10)	0.0% (*n* = 0)	0.0% (*n* = 0)	0.0% (*n* = 0)	0.0% (*n* = 0)	7.7% (*n* = 1)	0.0% (*n* = 0)	4.0% (*n* = 1)	21.0% (*n* = 4)	14.8% (*n* = 4)	7.4% (*n* = 2)

*Yes, performed; Partially, partially performed; No, not performed*.

*Due to the characteristics of the data, it was not possible to calculate differences between hospital- and community-based facilities, nor between geographical areas*.

### Visits and Examinations

From March to May 2020, only 28.4% of facilities all over the country continued to provide outpatient routine visits and examinations as usual, 59.4% provided visits but to a limited extent, while 12.2% ceased their activities. However, the majority of maternal and perinatal facilities were available for emergencies, either completely (68.8%) or to a limited extent (19.7%). All the facilities in which emergency visits were ceased were community-based and, except one, were located in Lombardy or Veneto. Regarding the waiting time for first visits and control visits, most centers reported that it was not extended (61.2 and 55.4%, respectively) or only partially (29.3 and 33.8%, respectively). Most of the facilities (68.8%; 78.3% prenatal vs. 54.8% postnatal, *p* = 0.03) had always or almost always kept fathers out of their partners' visits and exams.

Overall, at 24.7% of the facilities a part of the staff, and at 6.5% of the facilities, all the staff, continued their job in smart working mode. Most facilities continued to provide always, or almost always, in-person visits with physicians (82%), obstetricians (82.6%), and nurses (77.1%) during the pandemic. On the other hand, only 32.8% of facilities always or almost always provided in-person psychological visits. The facilities located in Lombardy or Veneto significantly more frequent in-person visits with physicians, compared to those located in the other regions, (91.3 and 74.1%, respectively, *p* = 0.04) and by psychologists (38.9 and 25.0%, respectively, *p* = 0.02). At the same time, obstetrician and nurse visits were significantly more frequently used by hospital-based professionals than by community-based colleagues (90.5 vs. 79.2%, *p* = 0.02 for obstetricians; 88.2 vs. 66.7%, *p* = 0.02 for nurses).

### Transformation Into a Dedicated COVID Facility

About a quarter of maternal or perinatal healthcare facilities (23.4%) were partially converted or transformed into COVID-19 units (16 out of 18 of these facilities were hospital-based) to provide care and support to the large number of patients infected by the severe acute respiratory syndrome coronavirus 2 (SARS-CoV-2). Further, two facilities (2.6%), that is, a hospital-based obstetrics and gynecology ward and a community-based birth center, were completely converted into COVID-19 facilities.

### Staff

Overall, a minority of facilities reported that some or all of the staff members (13 and 3.9%, respectively) were transferred to COVID-19 wards. This occurred significantly more frequently in hospital-based facilities than in community-based facilities. Nevertheless, almost one-fourth (23.4%) of the facilities, both hospital- and community-based, became understaffed during the index period due to various reasons such as ward transfer and sick leave.

About half of the facilities (46.0%) provided the entire staff with specific training on COVID-19 management, whereas a further 28.6% provided it only to select staff members. The remaining 25.4% did not provide any training.

Regarding the use of personal protective equipment (PPE) and the adoption of social/physical distancing, though perceived as essential and health-saving, both were considered very stressful by the staff of 68.2% of the facilities.

## Discussion

Our survey provides sobering insights into disruption to care and treatment for peripartum and perinatal patients (i.e., pregnant women, new mothers and their fetus/neonate) in Italy. We analyzed responses from 77 facilities in 11 Italian regions, covering relevant aspects of the activities and services provided in ante-, intra-, and post-partum clinical settings. Our data aligns with similar studies (6, 29) revealing that the pandemic has caused disruptions, with delays, reductions or cancellations in both maternal and neonatal appointments.

Regarding check-ups and examinations, although it is fully understandable that non-urgent services, such as many routine outpatient visits, were canceled in a well-intentioned effort to contain the spread of the new coronavirus (e.g., reports clearly show that, due to the pandemic, fewer women received follow-up care after obstetric anesthesia) (30). This change in access to medical and health services adversely affected the standard of maternal and perinatal care, including the realm of mental health care but particularly that of preventive, routine, and corrective medicine (5, 6, 29). The peripartum/perinatal population is particularly vulnerable, both physically and psychologically, to altered or delayed health care, because patients need and deserve close longitudinal monitoring (31). This is true for all pregnant and postpartum women as well as their babies because, for instance, even in case of a healthy young woman with non-complicated pregnancy (at least for a certain period), a complex maternal condition or fetal anomaly requiring multiple medical subspecialty consultations could occur. We must bear in mind that routine appointments are crucial to enable parents to participate in a shared decision-making process in all the cases in which there is uncertainty about medical conditions (32). Additionally, these consultations may also alleviate unnecessary parental anxiety. All these aspects must be considered when working during disasters such as the ongoing pandemic because, as highlighted by a systematic review on the effects of disaster on pregnancy and the postpartum period, they have an indirect impact on maternal mental health and some perinatal health outcomes (33). Moreover, it has been observed that the well-documented negative influence of mother's mental health on child development (34, 35) may be even greater after a disaster than any direct effect of disaster-related prenatal stress (33).

As regards telehealth (vs in-person check-ups), our data aligns with previous studies showing that it has been rapidly adopted in perinatal care since the onset of the pandemic (36–39). Telehealth offers safe access to consultation and follow-up appointments, saving patients both time and money, but is a complex system that normally requires years of implementation and optimization (40) in order to be an effective tool for providing comprehensive and multidisciplinary perinatal care, mainly in cases where physical examination is not or is rarely necessary. Face-to-face check-ups are still essential in high-risk cases (41). However, in certain cases, such as women with gestational diabetes mellitus, self-care programs *via* telemedicine may be a better choice than face-to-face visits (42).

In terms of healthcare workers, obstacles to effective care appear to include understaffing and additional stress for perinatal healthcare workers, and this aligns with the previously demonstrated increase in stress during the pandemic, stemming from staff shortages, excessive workload and the use of personal protective equipment (43). In terms of the patient's couple relationship, keeping patients (mothers and babies) together with their partner/other parent is crucial for respectful and effective care. However, consistently with other studies (5, 44, 45), our data show that partners/other parents are often excluded from the mother's check-ups and examinations in an effort to protect other patients and staff from infection.

As concerns the regional differences, our results indicate that facilities located in Lombardy or Veneto experienced a greater reduction in the provision of outpatient visits, especially emergency visits, and a statistically significant higher percentage of closures of community-based facilities. This is in line, on the one hand, with the Italian geographical distribution of the infection (northern regions faced disproportionately higher numbers of infections and deaths compared with southern and central Italy) (46), and on the other hand, with other Italian studies showing significant variations across regions in the way COVID-19 has affected medical specialist departments [e.g., radiology has changed during the pandemic with a large variability among different Italian regions; (47)]. With regard to the main difference between antenatal and postnatal services, that is, the degree to which fathers are permitted to attend their partners' visits and exams, one plausible and economical explanation is that different regions adopted different approaches to patient care, for instance different Italian regions implemented different strategies in terms of hospitalization, treatment in ICUs or home care for patients infected with SARS-CoV-2 (48). Here, we point out that Italian health system is regionally decentralized (thus, Italy has twenty regional health services), a situation that is not useful in controlling a pandemic, especially if we take into account the strong political pressure toward the transfer of tax resources from the central (national) government to the regions where income is produced (49).

Taken as a whole, the results of this study suggest that Italy was not entirely prepared to handle such a pandemic; indeed, specialist perinatal healthcare services have been (and still are) disrupted at many levels by this global health emergency. This is in line with other COVID-19 studies that have reported similar situations in other high-income countries (5, 29, 36–41, 43–45, 49–51). Our findings deepen the understanding of how the pandemic has influenced Italian healthcare facilities, and can be crucial in guiding the development and implementation of effective responses and, more broadly, in supporting and strengthening perinatal health systems. From this perspective, crises are also times of opportunity (12). The COVID-19 pandemic has caused us to rethink how to improve access to and implementation of perinatal healthcare services. The improvements forced by the current pandemic will be useful during the next phases as well as during future possible national or global health crises.

### Strengths and Limitations

The main strength of this research is that it is the first Italian study, and among very few international studies, that describe the effects of the COVID-19 pandemic on healthcare facilities and their provision of services. Thus, it may be helpful for the formulation of appropriate and evidence-based actions to be taken. However, in interpreting these results, certain limitations must be considered. First, the low response rate (5.4%) and of the fact that certain Italian regions are poorly represented or absent from the study. Thus, the results may not be representative of all perinatal healthcare facilities in Italy. However, it should be kept in mind that low response rates to online surveys in primary care are common and the extent to which results are affected is uncertain (52). Second, there is no information on the geographic location (urban vs. rural), patient volumes, and demographic characteristics of the responding facilities.

## Conclusion

The COVID-19 pandemic has disrupted maternal and perinatal healthcare activities and services, as well as increasing levels of stress among healthcare providers. This study sheds light on the effects of the COVID-19 pandemic on maternal and perinatal healthcare facilities and provides insights for policymakers. The management, allocation, and training of peripartum/perinatal healthcare workers can and must be improved. Italian policymakers and administrators are urged to work together to improve care for the most vulnerable. Prompt and continuous evaluation, along with timely and effective information on the status of healthcare facilities is fundamental to the development and implementation of contextually relevant guidelines.

## Data Availability Statement

The raw data supporting the conclusions of this article will be made available by the corresponding author upon request.

## Ethics Statement

The studies involving human participants were reviewed and approved by Ethics Committee of ASST Spedali Civili Hospital Brescia, Italy (NP4221 24.06.2020). The patients/participants provided their written informed consent to participate in this study.

## Author Contributions

LC and AS contributed equally to the general study design. LC and AT from the Observatory of Perinatal Clinical Psychology promote and coordinate the study in each health care service. MR, SC, and BM designed the plan of statistical analysis of the study. All authors have critically reviewed and agreed this final version of the article.

## Conflict of Interest

The authors declare that the research was conducted in the absence of any commercial or financial relationships that could be construed as a potential conflict of interest.
